# ETV7 promotes colorectal cancer progression through upregulation of IFIT3

**DOI:** 10.1007/s10142-023-01282-y

**Published:** 2024-01-10

**Authors:** Bao Chai, Yanjun Li, Yarong Guo, Zhuowei Zhang, Kai Jia, Xinhao Chai, Yuhong Suo

**Affiliations:** 1 Department of Gastroenterology, Shanxi Academy of Medical Science, Shanxi Bethune Hospital, Taiyuan, China; 2 Department of Surgery, Shanxi Academy of Medical Science, Shanxi Bethune Hospital, Taiyuan, China; 3 Department of Oncology, The First Affiliated Hospital of Shanxi Medical University, 85 South Jiefang Road, TaiyuanTaiyuan, 030001 Shanxi Province China; 4 Medical Imaging Department, Shanxi Medical University, Taiyuan, China; 5 Department of Surgery, The First Affiliated Hospital of Shanxi Medical University, Taiyuan, China; 6 Liver Cancer Center, Tianjin Medical University Cancer Institute and Hospital, Taiyuan, China

**Keywords:** Colorectal cancer, ETV7, IFIT3, Cancer progression

## Abstract

Members of the E26 transformation-specific (ETS) variant transcription factor family act as either tumor suppressors or oncogenic factors in numerous types of cancer. ETS variant transcription factor 7 (ETV7) participates in the development of malignant tumors, whereas its involvement in colorectal cancer (CRC) is less clear. In this study, The Cancer Genome Atlas (TCGA) and immunochemistry staining were applied to check the clinical relevance of ETV7 and interferon-induced protein with tetratricopeptide repeats 3 (IFIT3) in CRC patients. Overexpression and knockdown of ETV7 and IFIT3 were conducted by transfecting the cells with pCDNA3.1 plasmids and siRNAs, respectively. Western blotting was used to detect the protein expression of ETV7 in CRC cells. Cell Counting Kit-8, cell colony formation, and Transwell assays, as well as flow cytometry, were used to evaluate the proliferation, migration, cell cycle, and apoptosis of CRC cells. Furthermore, western blotting, RT-qPCR, and luciferase assay were used to explore the regulation of ETV7 on IFIT3. Rescue assay was used to investigate the significance of ETV7/IFIT3 axis on CRC progression. We found that ETV7 was upregulated in CRC tissues and cells. Overexpression of ETV7 stimulated the proliferation, migration, and cell cycle amplification, and reduced the apoptosis of CRC cells. Downregulation of ETV7 exerted the opposite effect on CRC cell progression. Moreover, we demonstrated that ETV7 stimulated the transcription activity, the mRNA and protein expression of IFIT3 in CRC cells. There was a positive correlation between ETV7 and IFIT3 in CRC patients. IFIT3 knockdown reversed the promotive effect exerted by overexpression of ETV7 on the amplification and migration of CRC cells. By contrast, overexpression of IFIT3 blocked the inhibitory effect of ETV7-targeting siRNA. In summary, ETV7 induces progression of CRC by activating the transcriptional expression of IFIT3. The EVT7/IFIT3 axis may be a novel target for CRC therapy.

## Introduction

Colorectal cancer (CRC) refers to a group of prevalent and lethal malignancies worldwide (Zhou et al. 2021). In 2020, > 1.9 million newly diagnosed cases of CRC and 935,000 related deaths occurred, representing approximately 10% of all cancer cases (Sung et al. 2021). Currently, numerous studies have identified the promising biomarkers that are vital factors in the diagnosis and treatment of CRC (Gu et al. 2023; Lech et al. 2016; Li et al. 2022). Understanding the molecular events triggering the progression of CRC can improve the efficacy of diagnosis and therapy for CRC patients.

Members of the E26 transformation-specific (ETS) gene family regulate a series of genes participating in various processes related to cancer, including cell amplification, migration, cell cycle regulation, and angiogenesis (De Braekeleer et al. 2012; Jiang et al. 2021; Wei et al. 2023). ETS variant transcription factor 7 (ETV7), also termed TEL2 or TELB, is a member of the ETS family. It is one of the 10% most upregulated genes in numerous types of cancer in humans (www.oncomine.org). Of note, elevated expression of ETV7 has been linked to B lymphomagenesis (Cardone et al. 2005), hematopoietic malignancy (Carella et al. 2006; Kawagoe et al. 2004), hepatocellular carcinoma (Matos et al. 2009), and bladder cancer (Li et al. 2021). Recent research investigated the tumorigenic functions of ETV7 through a mouse model of embryonal rhabdomyosarcoma. The investigators reported that ETV7 belonged to a rapamycin-insensitive mechanistic target of rapamycin kinase (mTOR) complex, which promoted tumor onset and accelerates tumor penetrance (Harwood et al. 2018). Subsequently, it was found that ETV7 controls the population of cancer stem cells in breast cancer and decreases the response to 5-fluorouracil along with radiotherapy (Pezze et al. 2021). ETV7 also mediates the resistance to doxorubicin through downregulation of DNAJ heat shock protein family (Hsp40) member C15 (DNAJC15) in breast cancer (Alessandrini et al. 2018). ETV7 regulates the immune microenvironment and, thus, is a poor prognostic factor for bladder cancer (Li et al. 2021). In contrast, it has been shown that ETV7 functions as a tumor suppressor in nasopharyngeal carcinoma by hindering serine protease inhibitor clade E member 1 (SERPINE1) expression (Sang et al. 2015). In nasopharyngeal carcinoma, ETV7 overexpression attenuates ectopic Snail expression or hypoxia-induced cell migration and invasion (Sang et al. 2018). ETV7 is downregulated in gastric cancer cells which are resistant to drugs and in biopsy specimens obtained after chemotherapy (Maeda et al. 2014). ETV7 expression is hampered in melanoma, potentially regulating the immune microenvironment; hence, ETV7 has been recognized as an independent and reliable predictor of prognosis in melanoma (Qu et al. 2020). Knockdown of ETV7 promotes the amplification, colony formation, and migration of ovarian cancer cells (Wu et al. 2020). Moreover, high expression of ETV7 is linked to a better prognosis (Huo et al. 2021). These studies suggest that whether ETV7 acts as an oncogenic or tumor suppressive protein is varied in different cancer types.

Interferon-induced protein with tetratricopeptide repeats 3 (IFIT3) was one of the four known IFIT family members in humans, which is an important component of the antiviral immune response and played significant roles in cancers (Tan et al. 2021). Franco et al. reported that IFIT3 promote metastasis in oral squamous cell carcinoma (Franco et al. 2023). IFIT3 silencing was sufficient to inhibit HCC cell migration, attenuating the aggressiveness of HCC cells (Liu et al. 2021). Besides that, IFIT3 could promote EGFR activation in OSCC cells and enhanced the tumor-preventive activity of gefitinib (Pidugu et al. 2019a, b). IFIT3 expression can also affect chemotherapy resistance in Pancreatic ductal adenocarcinoma (Wang et al. 2020a, b, c). However, the role of IFIT3 in CRC was unclear.

The aim of this study was to investigate the role and the underlying mechanisms of ETV7 in CRC. We showed that EVT7 acted as an oncogenic protein through upregulation of IFIT3. This knowledge could potentially provide a theoretical basis for the treatment of CRC.

## Materials and methods

### Analyzing the clinical relevance of ETV7 from The Cancer Genome Atlas (TCGA)

Data of a total of 275 colon adenocarcinoma samples and 349 adjacent samples were obtained from TCGA database. The expression of ETV7 in these samples was analyzed. The methylation level of the ETV7 promoter was calculated using data of 313 tumor samples and 37 normal samples obtained from TCGA database.

### Cell culture and transfection

Two CRC cell lines (i.e., RKO and HCT116) along with healthy control colorectal cells (NCM460) were cultured in Dulbecco’s modified Eagle’s medium (Gibco) supplemented with 10% fetal bovine serum, 100 µg/mL penicillin, and 100 µg/mL streptomycin (Invitrogen, Carlsbad, CA, USA). Cells were maintained in an incubator with 5% CO_2_ at a constant temperature of 37 °C.

The full-length coding sequence of ETV7 or interferon-induced protein with tetratricopeptide repeats 3 (IFIT3) was cloned into a pcDNA3.1 vector (Promega) by Hippobio Corporation (Huzhou, China) and verified through DNA sequencing. In addition, siRNAs were utilized to silence target mRNAs using LIPOFECTAMINE RNA-IMAX (Invitrogen) according to the instructions provided by the manufacturer. The siRNAs targeting ETV7 (siETV7 #1, 5′-CGCCACUAUUAUAAGCUUAAU-3′; siETV7 #2, 5′-GCAGGACAGAAUAGAGUUCAA-3′); siRNA targeting IFIT3 (siIFIT3 5′-GCGAUGUACCAUCUGGAUAAU-3′); and scrambled siRNA (siCtrl, 5′-CAGUACUUUUGUGUAGUACAAA-3′) were synthesized by Hippobio Corporation.

### Immunochemistry

Tumor samples and corresponding control samples were collected from patients with CRC who underwent surgery before any intervention, such as chemotherapy and radiotherapy, at the Shanxi Bethune Hospital (Taiyuan, China) between May 2018 and February 2021. The patients were histopathologically diagnosed by three independent pathologists. All patients provided written informed consent. This study was approved by the Ethics Committee of Shanxi Bethune Hospital (approval number: K-K0047) and carried out according to tenets stipulated in the Declaration of Helsinki of the World Medical Association.

Tissues sections were prepared (thickness: 5 μm), dried, and baked at 65 °C for 30 min. Citrate buffer was used for antigen retrieval at 100 °C for 15 min. The slides were incubated with methanol containing 3% hydrogen peroxide to block the endogenous peroxidase activity. Thereafter, the slides were incubated with anti-ETV7 antibody (Sigma, 1:200) at 4 °C overnight. This was followed by incubation with the corresponding secondary antibody (Vector Labs) for 30 min at 25 ℃. Images were captured using a Nikon E800 microscope.

### Cell proliferation assay

A Cell Counting Kit-8 (Beyotime, China) assay was used to assess cell viability. Briefly, cells were seeded into a 96-well plate (2 × 10^3^ cells/well, 200 μL/well) and treated with siRNAs targeting specific genes or overexpression plasmids and corresponding controls. After incubation for 1, 2, 3, and 4 days, 10 μL of CCK-8 solution was added to each well. After incubation for 1 h at 37 °C, the absorbance at 450 nm wavelength was examined using a microreader. The survival rate was calculated using the following formula: survival rate % = (OD treatment − OD blank)/(OD control − OD blank) × 100%.

### Cell colony formation assay

Single-cell suspensions of RKO or HCT116 cells were seeded in six-well plates (2 × 10^3^ cells/well) and cultured for 14 days. The medium was replaced every 3 days. Thereafter, phosphate-buffered saline was used to gently wash the cell colonies, and the colonies were fixed with 4% paraformaldehyde for 30 min at room temperature. Next, crystal violet solution (0.1%, Beijing Solarbio Science & Technology Co., Ltd.) was used to stain the colonies for 20 min at room temperature. Colonies were rinsed twice using phosphate-buffered saline to remove the excess stain and then counted and photographed. Colonies containing > 50 cells were counted.

### Transwell assay

Migration assays were conducted using Transwell chambers (Corning Inc., Corning, NY, USA). Initially, 4 × 10^4^ cells suspended in 200 μL of serum-free medium were seeded into the upper chambers of a 24-well Transwell plate, while the lower chamber was filled with medium supplemented with 10% fetal bovine serum. Subsequently, the cells were incubated at 37 °C for 1 day. Next, nonmigrating cells were gently removed, and the migrated cells in the lower filters were fixed with 4% polymethanol for 20 min and stained with crystal violet solution at a concentration of 0.1%. Finally, the number of migrated cells was counted under a microscope (Olympus FSX100).

### Reverse transcription-quantitative polymerase chain reaction (RT-qPCR)

RNA was extracted using the TRIzol reagent (Invitrogen). After detection of RNA concentration, 1 μg of total RNA was reverse transcribed into cDNA using SuperScript IV (Invitrogen). RT-qPCR was conducted using SYBR Green PCR Master Mix (Applied Biosystems) on a Roche LightCycler 480 II PCR instrument (Basel, Switzerland). The thermal cycling profiles for qRT-PCR were heating at 95 °C for 30 s, then 40 cycles at 95 °C for 5 s and 60 °C for 30 s. Glyceraldehyde-3-phosphate dehydrogenase (GAPDH) was used as the internal control. Data for the expression of indicated genes were collected and quantitatively analyzed with the 2^−∆∆Ct^ method. The following primers were used in this experiment: IFIT3-forward, 5′-AAAAGCCCAACAACCCAGAAT-3′; IFIT3-reverse, 5′-CGTATTGGTTATCAGGACTCAGC-3′; GAPDH-forward, 5′-GGAGCGAGATCCCTCCAAAAT-3′; and GAPDH-reverse, 5′-GGCTGTTGTCATACTTCTCATGG-3′.

### Western blotting

Cells were primarily lysed in 1 × lysis buffer supplemented with 1 mM phenylmethylsulfonyl fluoride (Cell Signaling Technology, Danvers, MA, USA). The concentration was determined using the BCA assay kit (Bio-Rad). Protein (20 µg) was loaded to each lane of precast 12% polyacrylamide gel electrophoresis gels and subjected to electrophoresis. Thereafter, the proteins were transferred onto a nitrocellulose membrane through the Bio-Rad system (Bio-Rad). The membranes were blocked using a 5% milk solution and incubated with the following primary antibodies at 4 °C overnight: anti-ETV7 (1:1,000; Sigma); anti-IFIT3 (1:1,000; Proteintech); and β-actin (1:1,000; Proteintech). Subsequently, the membranes were incubated with secondary horseradish peroxidase-conjugated antibody (1:5000, Pierce) in tris-buffered saline with Tween 20 at room temperature for 1 h. The expression of targeted proteins was visualized using Femto Chemiluminescent Substrate (Pierce).

### Cell apoptosis

Fluorescein isothiocyanate (FITC) Annexin V/propidium iodide (Annexin V/PI) Apoptosis Detection Kit I (BD Biosciences) was utilized to determine the degree of cell apoptosis. Briefly, transfected cells in the logarithmic growth phase were harvested and washed with 1 × binding buffer. Cells (1.5 × 10^5^) were stained with FITC Annexin V (5 μL) and PI (2 μL). Next, 1 × Annexin V Binding Buffer (400 μL) was added to each sample, and the samples were analyzed using a CytoFLEX S flow cytometer and CytExpert 2.3 software (Beckman Coulter, Inc.).

### Cell cycle analysis

A total of 2.5 × 10^5^ CRC cells were seeded into a 6-well plate. After culturing for 24 h, the cells were transfected with corresponding plasmids or siRNAs. 48 h later, the CRC cells in the logarithmic growth phase were harvested and mixed with 70% ethanol at − 20 °C overnight. Thereafter, the cells were rinsed and incubated with 20 μg/mL RNase A as well as 5 μL of propidium iodide (PI, 10 µg/mL, Sigma-Aldrich, St Louis, MO, USA) for 30 min at 37 °C in the dark. The cell cycle was examined using flow cytometry.

### IFIT3 promoter luciferase assay

The IFIT3 promoter region was cloned into a pGL 3.1 luciferase reporter vector (pGL 3.1-IFIT3; Promega). HCT116 or RKO cells (1.5 × 104 cells/well) were seeded into a 24-well plate in triplicate. CRC cells were co-transfected with 400 ng pGL 3.1-IFIT3, 100 ng pRL-TK vectors (normalization control), pcDNA3.1 plasmids (empty control or ETV7 overexpression), or 100 nM siRNAs (siControl or siETV7) using LIPOFECTAMINE and RNA-IMAX (Invitrogen). After transfection for 48 h, the luciferase activity in cells was detected using the Dual-Luciferase Reporter Assay System (Promega). Firefly luciferase activities were normalized to Renilla luciferase activities.

### Statistical analysis

GraphPad Prism version 8.0 software (GraphPad Software Inc., San Diego, CA, USA) was used for statistical analysis. Student’s *t*-test was used to analyze differences between two groups. *P* values < 0.05 indicate statistically significant differences.

## Results

### ETV7 was highly expressed in CRC tissues and cells

**Fig. 1:**
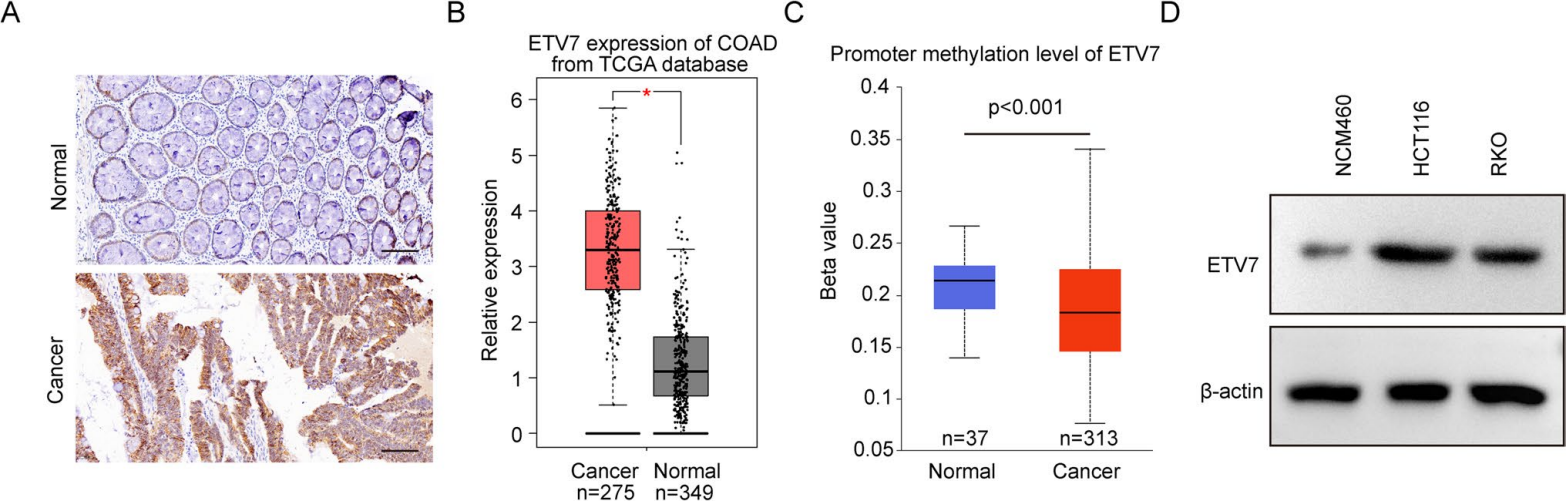
ETV7 was highly expressed in CRC. **A** CRC tissues (*n* = 20) and paracancerous (*n* = 20) tissues were subjected to immunochemistry staining of ETV7. **B** ETV7 expression in CRC (*n* = 275) and control samples (*n* = 349) based on the information from TCGA database. **C** Methylation level of the ETV7 promoter in CRC (*n* = 313) and control samples (*n* = 37) based on the information from TCGA database. **D** The protein expression of ETV7 was checked in two types of CRC cells (RKO and HCT116) and normal colorectal cells (NCM460)

The levels of ETV7 in patients with CRC were examined using immunohistochemical staining from a CRC tissue microarray. ETV7 expression was higher in cancer versus normal samples (Fig. 1A and Table 1). Data from TCGA revealed high ETV7 mRNA expression and low ETV7 promoter methylation (Fig. 1B, C). In addition, we analyzed the correlation between ETV7 and clinical features in patients with CRC. However, the results showed that there are no significantly between ETV7 and age, gender, pathologic T stage, N stage, and M stage (Table 2). ETV7 was significantly overexpressed in RKO and HCT116 cells compared with NCM460 cells (Fig. 1D). These results indicated high ETV7 expression in both CRC tissues and cells, as well as low methylation of the ETV7 promoter.

**Table 1:** The expression of ETV7 in normal tissue and CRC tissue by IHC

	Tumor tissue	Adjacent tissue	*χ*^2^	*P* value
ETV7 high expression	15	8	5.012	0.025
ETV7 low expression	5	12		
Total	20	20

**Table 2:** Correlation of ETV7 expression and clinical features in patients with CRC

Characteristics	Low expression of ETV7	High expression of ETV7	*P* value
*n*	322	322	
Age, *n* (%)			0.633
≤ 65	141 (21.9%)	135 (21%)	
> 65	181 (28.1%)	187 (29%)	
Gender, *n* (%)			0.097
Female	161 (25%)	140 (21.7%)	
Male	161 (25%)	182 (28.3%)	
Pathologic T stage, *n* (%)			0.654
T1	10 (1.6%)	10 (1.6%)	
T2	50 (7.8%)	61 (9.5%)	
T3	224 (34.9%)	212 (33.1%)	
T4	35 (5.5%)	39 (6.1%)	
Pathologic N stage, *n* (%)			0.292
N0	184 (28.7%)	184 (28.7%)	
N1	69 (10.8%)	84 (13.1%)	
N2	65 (10.2%)	54 (8.4%)	
Pathologic M stage, *n* (%)			0.069
M0	233 (41.3%)	242 (42.9%)	
M1	53 (9.4%)	36 (6.4%)	

### ETV7 promoted the amplification and migration of CRC cells

**Fig. 2:**
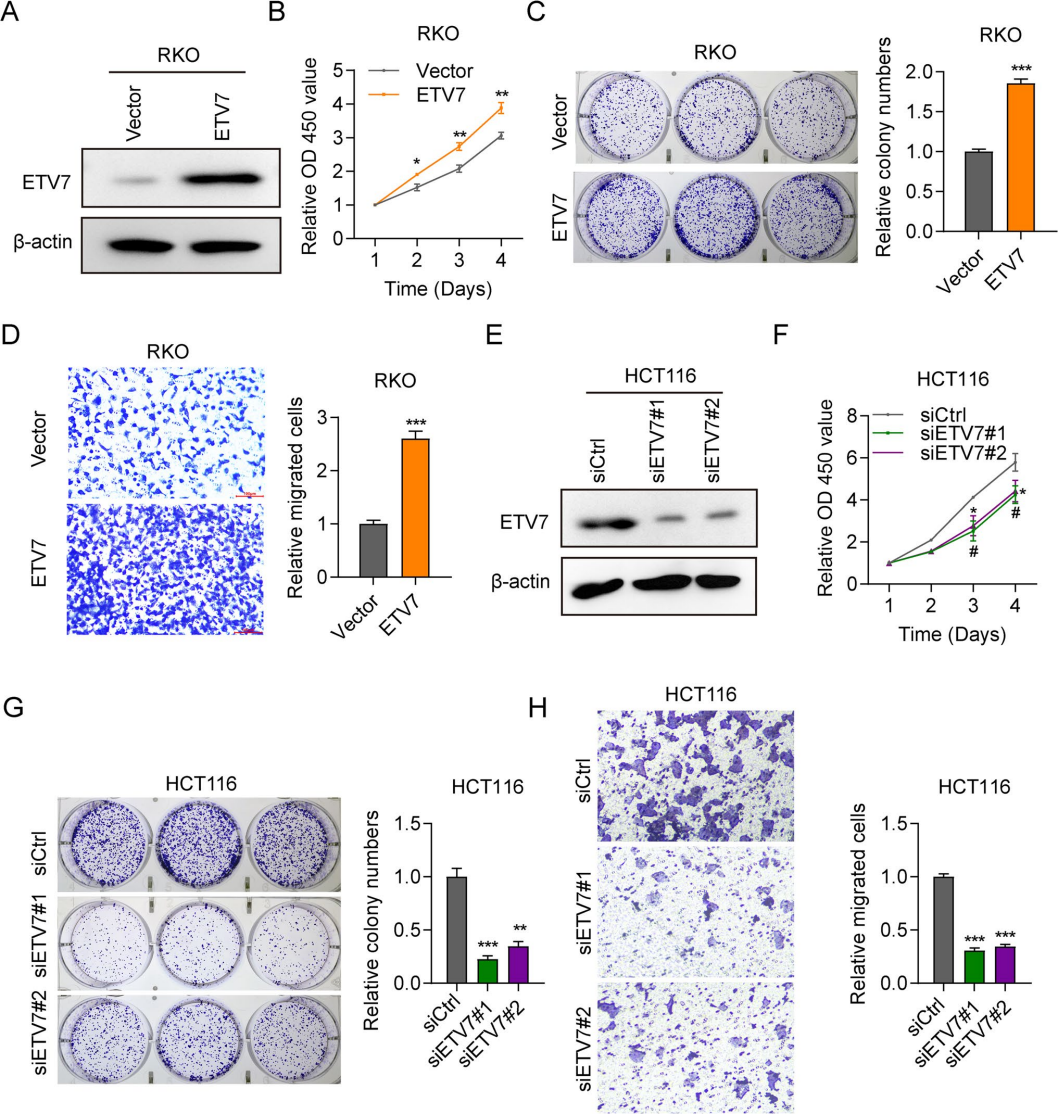
ETV7 promoted the amplification and migration of CRC cells. **A** The protein expression of ETV7 was checked in RKO cells transfected with pcDNA empty vector (vector) or pcDNA-ETV7 (ETV7). **B** Cell viability was measured in RKO cells transfected with pcDNA empty vector (vector) or pcDNA-ETV7 (ETV7). *n* = 3. **C, D** Cell colony formation and migration ability were examined in RKO cells transfected with pcDNA empty vector (vector) or pcDNA-ETV7 (ETV7). *n* = 3. **E** The protein expression of ETV7 was checked in siCtrl, siETV7#1, and siETV7#2 HCT116 cells. **F** Cell viability was measured in siCtrl, siETV7#1, and siETV7#2 HCT116 cells. *n* = 3. **G, H** Cell colony formation and migration ability were examined n siCtrl, siETV7#1, and siETV7#2 HCT116 cells. *n* = 3. *p < 0.05; **p < 0.01; ***p < 0.001

RKO or HCT116 cells were transfected with pcDNA-ETV7 or siETV7, and the efficiency of overexpression or knockdown was examined by western blotting (Fig. 2A, E). The viability of RKO cells was increased in the ETV7 overexpression group compared with the Control group (Fig. 2B). The viability of HCT116 cells was decreased in the ETV7 knockdown group compared with the siControl group (Fig. 2F). We also assessed the colony-forming capacity of transfected CRC cell lines. In RKO cells, the number of colonies was higher for cells with upregulated ETV7 expression versus Control cells (Fig. 2C). In HCT116 cells, the number of colonies was lower for cells with downregulated ETV7 expression versus cells treated with siControl (Fig. 2G). ETV7 overexpression also increased the migration of RKO cells (Fig. 2D), while siETV7 decreased the migration of HCT116 cells (Fig. 2H). Based on these results, ETV7 expression appeared to confer a growth advantage in CRC cell lines; thus, it may function as an oncogenic factor.

### ETV7 reduced cell apoptosis and cell cycle arrest of CRC cells

**Fig. 3:**
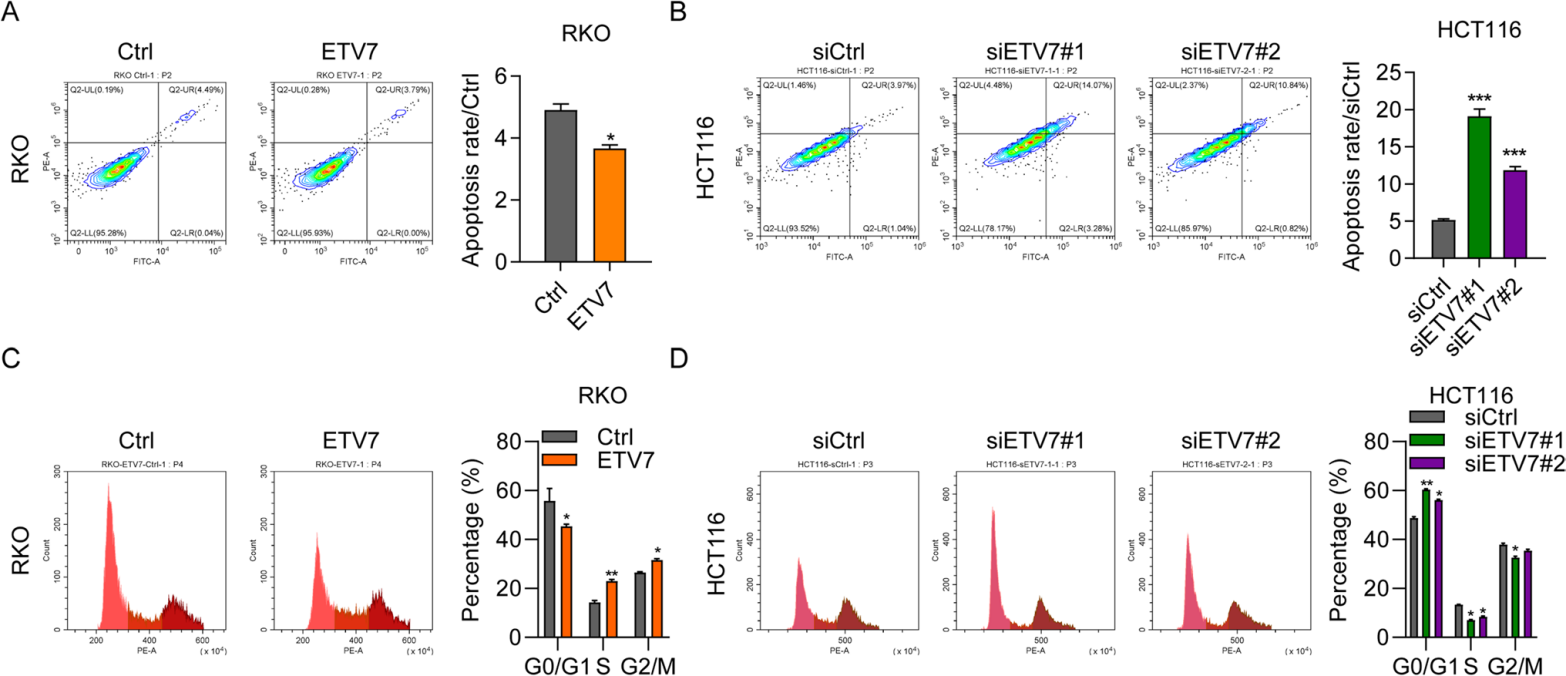
ETV7 reduced apoptosis and cell cycle arrest in colorectal cancer cells. **A** Rate of apoptosis in RKO cells transfected with pcDNA empty vector (vector) or pcDNA-ETV7 (ETV7). *n* = 3. **B** Rate of apoptosis in siCtrl, siETV7#1, and siETV7#2 HCT116 cells. *n* = 3. **C** Cell cycle distribution of RKO cells transfected with pcDNA empty vector (vector) or pcDNA-ETV7 (ETV7). *n* = 3. **D** Cell cycle distribution of siCtrl, siETV7#1, and siETV7#2 HCT116 cells. *n* = 3. *p < 0.05; **p < 0.01; ***p < 0.001

ETV7 has been mainly associated with cell apoptosis (Li et al. 2018). Flow cytometry results revealed lower levels of apoptosis in pcDNA-ETV7-treated RKO cells compared with Control cells (Fig. 3A). In addition, the level of apoptosis was higher in siETV7-treated HCT116 cells versus siControl-treated cells (Fig. 3B). ETV7 overexpression promoted cell cycle progression in CRC cells by decreasing the number of cells in the G0/G1 phase (Fig. 3C). In contrast, siETV7 caused cell cycle arrest in the G0/G1 phase (Fig. 3D). This evidence suggested that ETV7 regulated the apoptosis and cell cycle progression of CRC cells in vitro.

### ETV7 positively regulated the expression of IFIT3

**Fig. 4:**
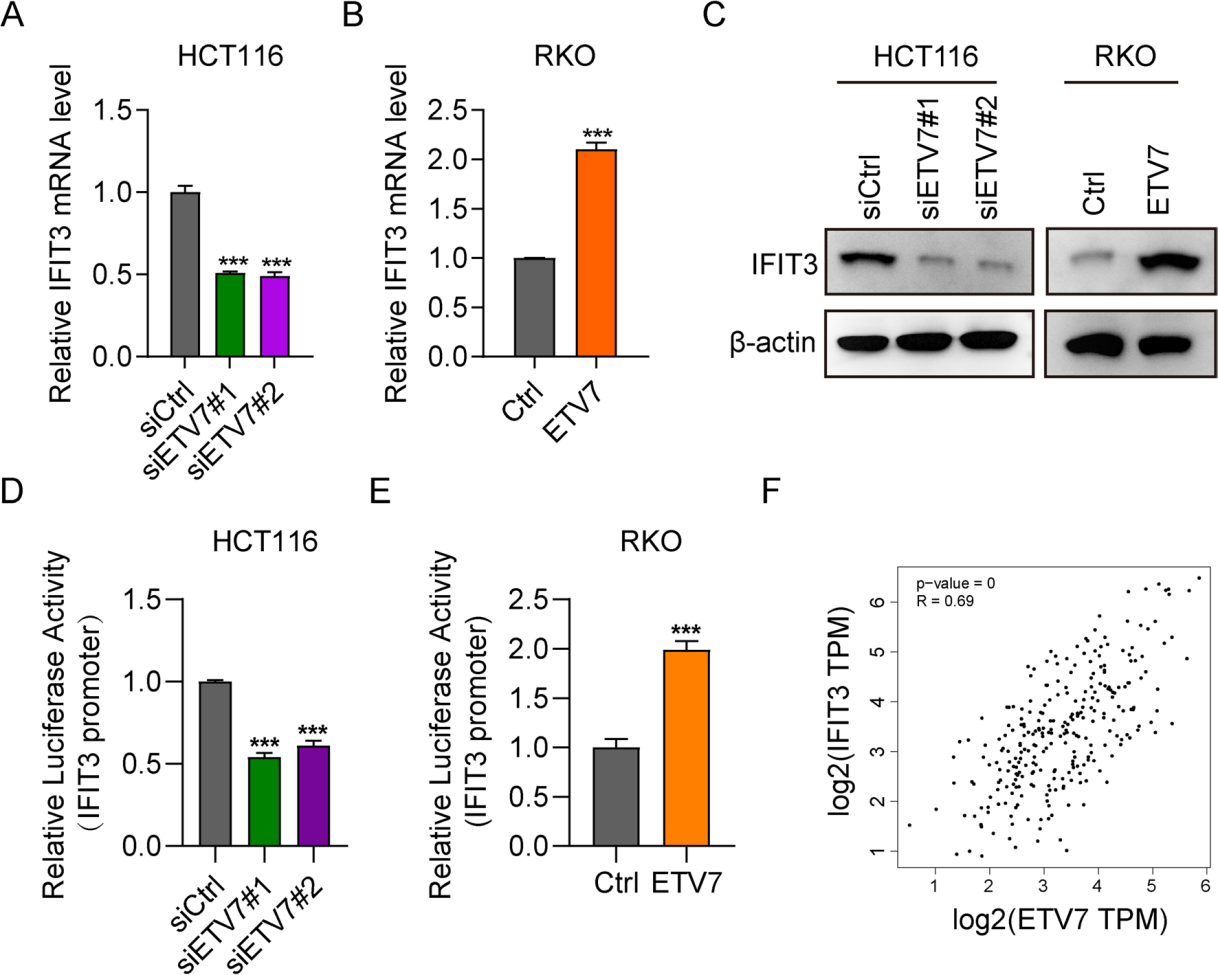
ETV7 positively regulated the expression of IFIT3. **A–C** The qPCR (*n* = 3) and WB analyses detected IFIT3 expression in RKO cells transfected with pcDNA-ETV7 and HCT16 cells transfected with siETV7. **D, E** IFIT3 promoter luciferase activity was detected in RKO and HCT16 cells 24 h following transfection with pcDNA-ETV7 or siRNAs targeting ETV7. *n* = 3. **F** Pearson’s correlation of ETV7 and IFIT3 expression using data from TCGA database. ***p < 0.001

Using data from TCGA database, we analyzed the genes which exhibit a positive correlation with ETV7 in CRC tissues. IFIT3 (an established oncogene) was among the top 10 genes. Previous studies have demonstrated that IFIT3 acted as an oncogene and could be upregulated by various factors (Xu et al. 2019; Zan et al. 2022). However, the regulation of ETV7 on IFIT3 remained unknown in CRC. Thus, we examined whether ETV7 regulated IFIT3 expression. The results showed that knockdown of ETV7 reduced the mRNA and protein expression of IFIT3, whereas overexpression of ETV7 increased the levels of IFIT3 (Fig. 4A–C). Furthermore, ETV7 siRNAs significantly downregulated the IFIT3 promoter-driven luciferase activity, which was upregulated by constitutive overexpression of ETV7 (Fig. 4D, E). We also uncovered a positive correlation between ETV7 and IFIT3 (*R* = 0.69) in CRC samples based on TCGA database (Fig. 4F). These data indicated that ETV7 positively regulates IFIT3 expression in CRC.

### IFIT3 knockdown suppressed the cellular function in ETV7-overexpressing CRC cells

**Fig. 5:**
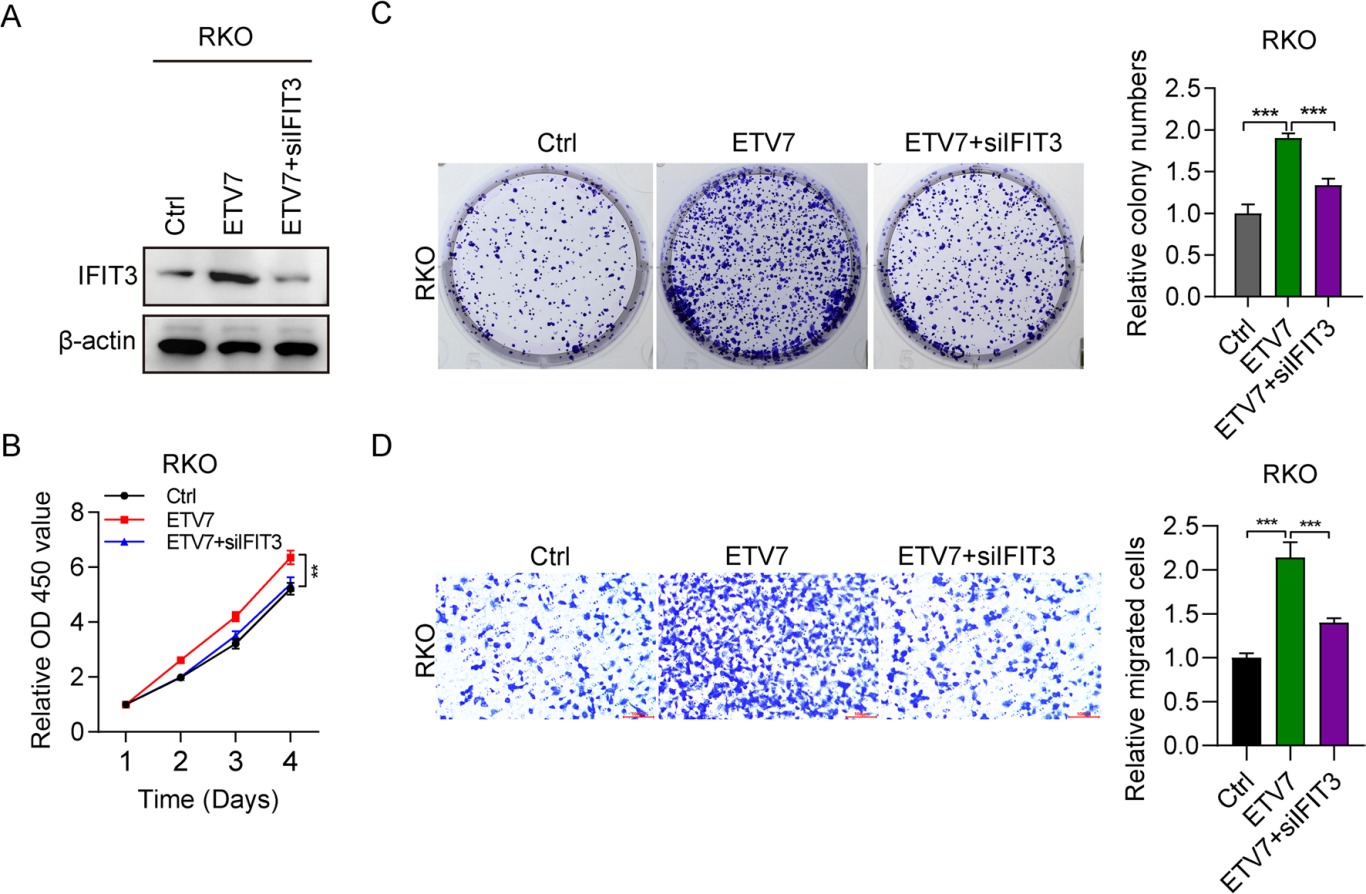
IFIT3 knockdown inhibited the cellular function in ETV7-overexpressing cells. **A** The protein expression of IFIT3 in RKO cells transfected with pcDNA-ETV7 and pcDNA-ETV7 + siIFIT3. **B** Viability of RKO cells transfected with pcDNA-ETV7 and pcDNA-ETV7 + siIFIT3. *n* = 3. **C** Colony numbers of RKO cells transfected with pcDNA-ETV7 and pcDNA-ETV7 + siIFIT3. *n* = 3. **D** Numbers of migrated RKO cells transfected with pcDNA-ETV7 and pcDNA-ETV7 + siIFIT3. *n* = 3. **p < 0.01; ***p < 0.001

We sought to identify whether ETV7-mediated cancer progression is based on the expression of IFIT3. Therefore, we knocked down IFIT3 expression with siRNA in RKO cells, in which ETV7 was overexpressed. The protein expression of IFIT3 was markedly increased after EVT7 overexpression, whereas it was significantly decreased following treatment with siIFIT3 (Fig. 5A). The use of siIFIT3 also reduced the cell viability, which had been promoted by ETV7 overexpression, to almost normal levels (Fig. 5B). All ETV7-mediated effects on cell colony formation and migration were abolished by treatment with siIFIT3 (Fig. 5C, D). These outcomes indicated that IFIT3 participated in the ETV7-mediated development of CRC.

### IFIT3 overexpression restored the cellular function in ETV7-silenced CRC cells

**Fig. 6:**
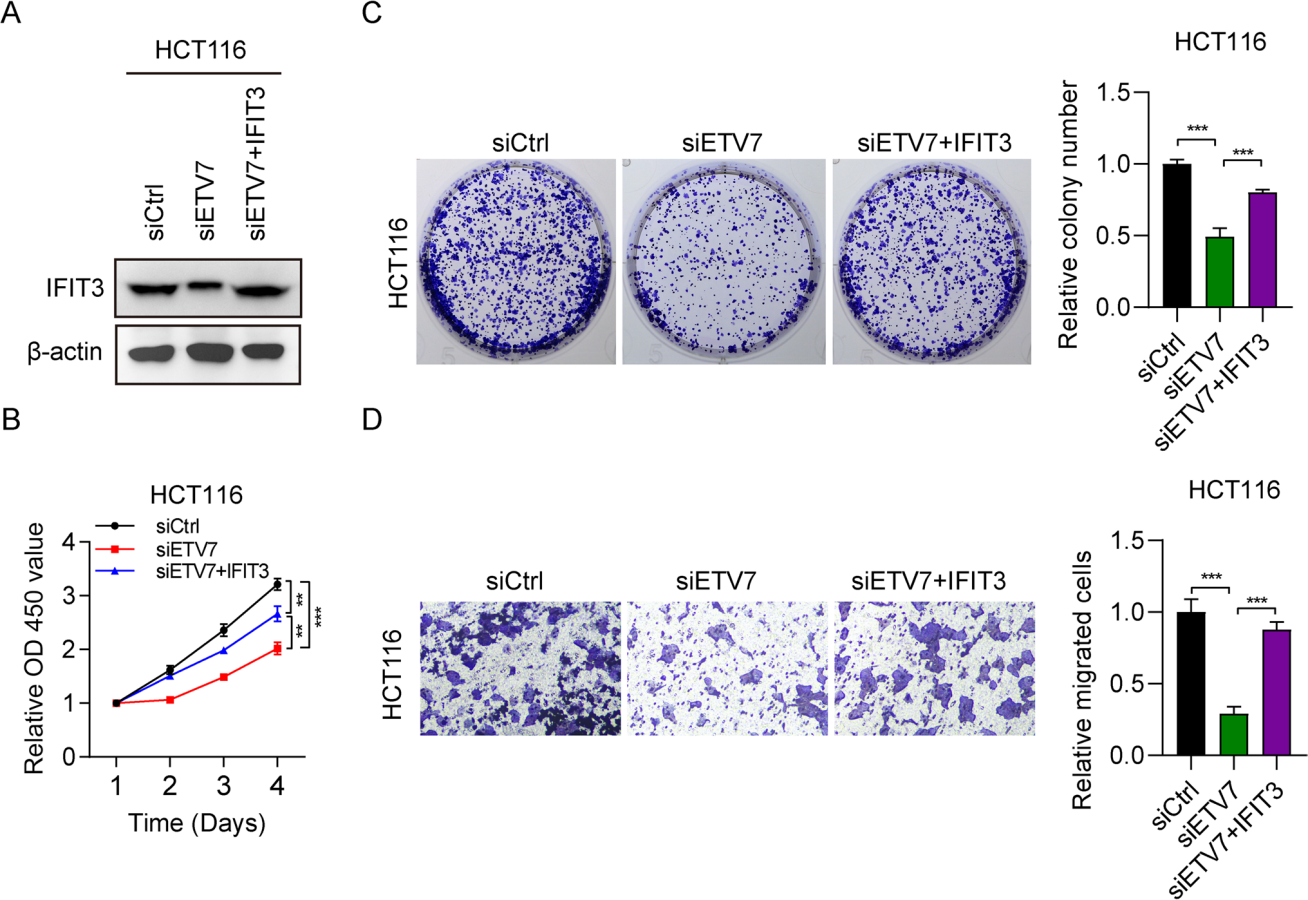
IFIT3 overexpression rescued the cellular function in ETV7-silenced colorectal cancer cells. **A** IFIT3 expression in HCT116 cells transfected with siETV7 and siETV7 + pcDNA-IFIT3 was detected using WB. **B** Viability of HCT116 cells transfected with siETV7 and siETV7 + pcDNA-IFIT3. *n* = 3. **C, D** Colony numbers and numbers of migrated HCT116 cells transfected with siETV7 and siETV7 + pcDNA-IFIT3. *n* = 3. **p < 0.01; ***p < 0.001

Furthermore, we also overexpressed IFIT3 in HCT116 cells transfected with siETV7. The protein levels of IFIT3 were significantly decreased after EVT7 knockdown, whereas they were obviously increased following IFIT3 overexpression (Fig. 6A). The siETV7-mediated effects on the proliferation and colony growth of HCT116 cells were recovered after IFIT3 overexpression (Fig. 6B, C). Transwell assay also yielded similar results (Fig. 6D). These findings confirmed that siETV7 suppressed cell growth and migration by targeting IFIT3.

## Discussion

Due to its high morbidity and mortality rates, cancer has always been a great threat to human health (Zuo and Kwok 2021). When the balance of either an increase in cell proliferation or a decrease in cell death is disturbed, cancers can occur (Gerl and Vaux 2005). Cell cycle is a conserved mechanism that eucaryotic cells replicate themselves by using or controlling a shared a set of factors(King and Cidlowski 1995). During cell cycle checkpoints that occur at the G1/S boundary, in S-phase, and during the G2/M-phases, the timing and order of cell cycle events are monitored (MacLachlan et al. 1995). Apoptosis is also a highly conserved mechanism which enables an organism to eliminate unwanted and defective cells commit suicide, without causing undesirable inflammatory response (Jacobson et al. 1997). Therefore, targeting the genes involved in cell cycle or apoptosis effective methods for cancer therapy.

The ETS transcription factor family is implicated in cellular amplification and apoptosis (Maroulakou and Bowe 2000). ELK3, as a type of ETS protein, was shown to promote cell cycle, proliferation, and EMT, and suppressed cell apoptosis, which could be a promising diagnostic and prognostic biomarker in human cancer(Al-Hawary et al. 2023). Yan et al. reported that overexpression of ELK1 increased pancreatic cancer cells proliferation, invasion, and survival (Yan et al. 2021). Tan et al. revealed that ETS2 silencing reduced cardiomyocyte apoptosis and autophagy to slow Heart failure (HF) progression(Tan et al. 2023).

In CRC, the functions of ETS family members differ. For example, ETV1, ETV5, ETV4, EHF, ELK1, ETL4, ETS1, ETS2, and ERG act as tumor promoters accelerating angiogenesis in CRC (Cheng et al. 2019; Li et al. 2016; Ma et al. 2021a, b; Meng et al. 2019; Wang et al. 2020a, b, c; Wang et al. 2020a, b, c; Yao et al. 2021; Zhu et al. 2020). In contrast, SPIB, SPDEF, and ELF5 exert anti-CRC effects (Ma et al. 2021a, b; Noah et al. 2013; Zhao et al. 2021). In patients with lymph node metastasis of CRC, ETV4 expression was significantly upregulated, whereas SPDEF and SPIB expression was significantly downregulated (Deves et al. 2011). However, the role of ETV7 in CRC was not clear.

In this study, we found that ETV7 expression was elevated in CRC. The methylation of ETS family members has been associated with cancer progression (Schulz and Hatina 2006). In this analysis, we also found a low level of ETV7 promoter methylation in CRC cells. Furthermore, we demonstrated that ectopic expression of EVT7 exerted a promotive effect on the amplification, migration, and cell cycle progression in CRC cells. In contrast, knockdown of EVT7 induced opposite effects, indicating the tumor-promoting role of ETV7 in CRC progression. The results of Annexin V/PI staining suggested that ETV7 also inhibits the apoptosis of CRC cells. These findings are consistent with previous evidence (Carella et al. 2006); nonetheless, the functions of ETV7 in different tumors remain controversial. The limitation of the in vitro study was the certain differences between the cultured samples and the in vivo environment. Therefore, in the next study, we will focus on the clinal samples and animal experiments to discuss the function of ETV7 on CRC.

Emerging studies have identified that IFIT3 is involved in cell proliferation, apoptosis, differentiation, and cancer development, and it also could be a potential biomarker in disease diagnosis and therapy (Pidugu et al. 2019a, b; Zhang et al. 2023). In colon cancer cells (i.e., HT29 and HCT116), IFIT3 expression was downregulated under treatment with the anti-programmed cell death-1 (anti-PD-1) antibody nivolumab (Ierano et al. 2022). However, it was upregulated in absence of melanoma 2 (AIM2)-overexpressing (induced by interferon-gamma [IFN-γ]) CRC cell lines (Lee et al. 2012). The function of IFIT3 in CRC was not been reported yet.

In this study, using data from TCGA database, we confirmed that the expression of IFIT3 was positivity correlated with ETV7. Luciferase assay results showed that the IFIT3 promoter could be regulated by ETV7. Moreover, recovery assays also demonstrated that treatment with siIFIT3 reduced cell viability and migration, which had been promoted by ETV7 overexpression. In contrast, IFIT3 upregulation recovered the cell viability and migration that had been decreased by treatment with siETV7. These results revealed that ETV7 promoted CRC progression through the regulation of IFIT3. The limitation of our study is that the deep study between the relationship of ETV7 and IFIT3 in clinical samples were not discussed, and the clinical significance was unclear. Besides, the molecular binding mechanism between also ETV7 and IFIT3 also need to be explored in CRC. Furthermore, the recovery experiments should be carried out in vivo experiments with animal model.

## Conclusion

The results of the present study indicated that the ETV7–IFIT3 axis may facilitate tumorigenesis in CRC. Interference with ETV7 or IFIT3 may be a strategy for hindering the proliferation and migration of CRC cells.

## Data Availability

Data and materials generated are relevant to the results that are included in this article. Other data are available from the corresponding author upon reasonable request.

## Abbreviations

ETV7: ETS variant transcription factor 7
CRC: Colorectal cancer
ETS: E26 transformation-specific
IFIT3: Interferon-induced protein with tetratricopeptide repeats 3
COAD: Colon adenocarcinoma
TCGA: The Cancer Genome Atlas
DMEM: Dulbecco’s modified Eagle’s medium
